# An omniphobic lubricant-infused coating produced by chemical vapor deposition of hydrophobic organosilanes attenuates clotting on catheter surfaces

**DOI:** 10.1038/s41598-017-12149-1

**Published:** 2017-09-14

**Authors:** Maryam Badv, Iqbal H. Jaffer, Jeffrey I. Weitz, Tohid F. Didar

**Affiliations:** 1 School of Biomedical Engineering, McMaster University, Hamilton, Ontario Canada; 2 Thrombosis & Atherosclerosis Research Institute (TaARI), Hamilton, Ontario Canada; 3 Department of Surgery, McMaster University, Hamilton, Ontario Canada; 4 Department of Medicine and Biochemistry and Biomedical Sciences, McMaster University, Hamilton, Ontario Canada; 5 Department of Mechanical Engineering, McMaster University, Hamilton, Ontario Canada

## Abstract

Catheter associated thrombosis is an ongoing problem. Omniphobic coatings based on tethering biocompatible liquid lubricants on self-assembled monolayers of hydrophobic organosilanes attenuate clotting on surfaces. Herein we report an efficient, non-invasive and robust process for coating catheters with an antithrombotic, omniphobic lubricant-infused coating produced using chemical vapor deposition (CVD) of hydrophobic fluorine-based organosilanes. Compared with uncoated catheters, CVD coated catheters significantly attenuated thrombosis via the contact pathway of coagulation. When compared with the commonly used technique of liquid phase deposition (LPD) of fluorine-based organosilanes, the CVD method was more efficient and reproducible, resulted in less disruption of the outer polymeric layer of the catheters and produced greater antithrombotic activity. Therefore, omniphobic coating of catheters using the CVD method is a simple, straightforward and non-invasive procedure. This method has the potential to not only prevent catheter thrombosis, but also to prevent thrombosis on other blood-contacting medical devices.

## Introduction

Blood-contacting medical devices such as catheters, heart valves and vascular grafts are widely used. All such devices are prone to thrombosis, which can lead to thromboembolic complications and device failure^1^. Cancer patients often have central venous catheters implanted for venous access and for parenteral delivery of chemotherapy, antibiotics and nutrition. Catheter thrombosis is common in these patients and can lead to deep-vein thrombosis and pulmonary embolism; complications that often delay treatment, extend hospital stay and increase healthcare costs^2^. Therefore, methods to reduce catheter thrombosis are worthwhile.

Thrombosis on catheters and other blood-contacting medical devices is a multi-step process that starts with adhesion of proteins and cells, and culminates in the formation of a platelet-fibrin mesh^3,4^. Coagulation on these surfaces is activated via the contact pathway, which is initiated by the adsorption and activation of factor (F) XII^5^. Therefore, attenuation of thrombosis on medical devices requires processes that prevent activation of the contact pathway. Such processes can be active or passive. Active processes designed to limit contact activation include surface coating with heparin^6^, which inhibits multiple steps in blood coagulation, or with corn trypsin inhibitor^7^, a potent and specific inhibitor of FXIIa. Passive processes to retard contact activation include surface modifications with synthetic or natural polymers and biomolecules^8–10^ such as poly (ethylene oxide) (PEO)^11–15^ and polyethylene glycol (PEG)^16–18^, poly-sulfobetaine^19,20^, poly-2-methoxyethyl acrylate (PMEA)^21^, and albumin^22–27^. More recently, omniphobic lubricant-infused coatings have been developed based on tethering biocompatible, perfluorocarbon lubricants on self-assembled monolayers (SAMs) of hydrophobic organosilanes^28,29^. Based on fluorous chemistry, fluorous molecules can be physically adsorbed onto fluorous-containing surfaces^30^. The strong intermolecular interaction between the fluorinated lubricant and the fluorosilane layer locks the lubricant liquid onto the surface, thereby creating a highly stable, omniphobic lubricant-infused coating^29^. These surfaces outperform heparin-coated surfaces, as well as a range of hydrophilic coatings^19^ developed to resist blood clot formation. Furthermore, lubricant-infused omniphobic coatings have been more effective than PEG or albumin for blocking non-specific adhesion of cells and bacteria^31,32^. In addition to increasing blood-compatibility, these surfaces are stable and durable when exposed to physiological shear stress *in vitro* ^33,34^. Therefore, lubricant-based omniphobic coating of biomedical devices is a promising method for preventing thrombus formation.

Lubricant-based omniphobic coatings are produced by applying SAMs of hydrophobic organosilane (*e.g*. tridecafluoro-1,1,2,2-tetrahydrooctyl trichlorosilane) onto the surface. Liquid phase deposition (LPD) is the main technique reported in the literature for producing SAMs of fluorine-based silanes in order to obtain lubricant-infused omniphobic coatings^32^. However, the LPD method has several limitations. First, the high volumes of solvent waste produced during the procedure are harmful to the environment, which restricts the industrial viability of the process^35^. Second, self-polymerization of silanes in the solution phase may impair the formation of homogenous silane layers on the surface^36^. Third, and most important, surfaces treated by LPD are exposed to the impurities and side products produced during the treatment process, which may compromise the material and alter the bulk properties of its surface^35^. Such alterations are particularly problematic for materials used for biomedical applications.

To overcome the limitations of LPD, we set out to develop a more robust, simplified and clinically relevant chemical vapor deposition (CVD) method for creating a lubricant-infused omniphobic coating on FDA-approved catheters. The surface properties, chemical composition and antithrombotic activity of catheters coated in this manner were compared with those of uncoated catheters and catheters coated using the LPD method. We show that the CVD method has less of an effect on the surface topography of catheters than the LPD method and endows them with greater antithrombotic activity.

## Results

### Producing omniphobic lubricant-infused catheters

**Figure 1:**
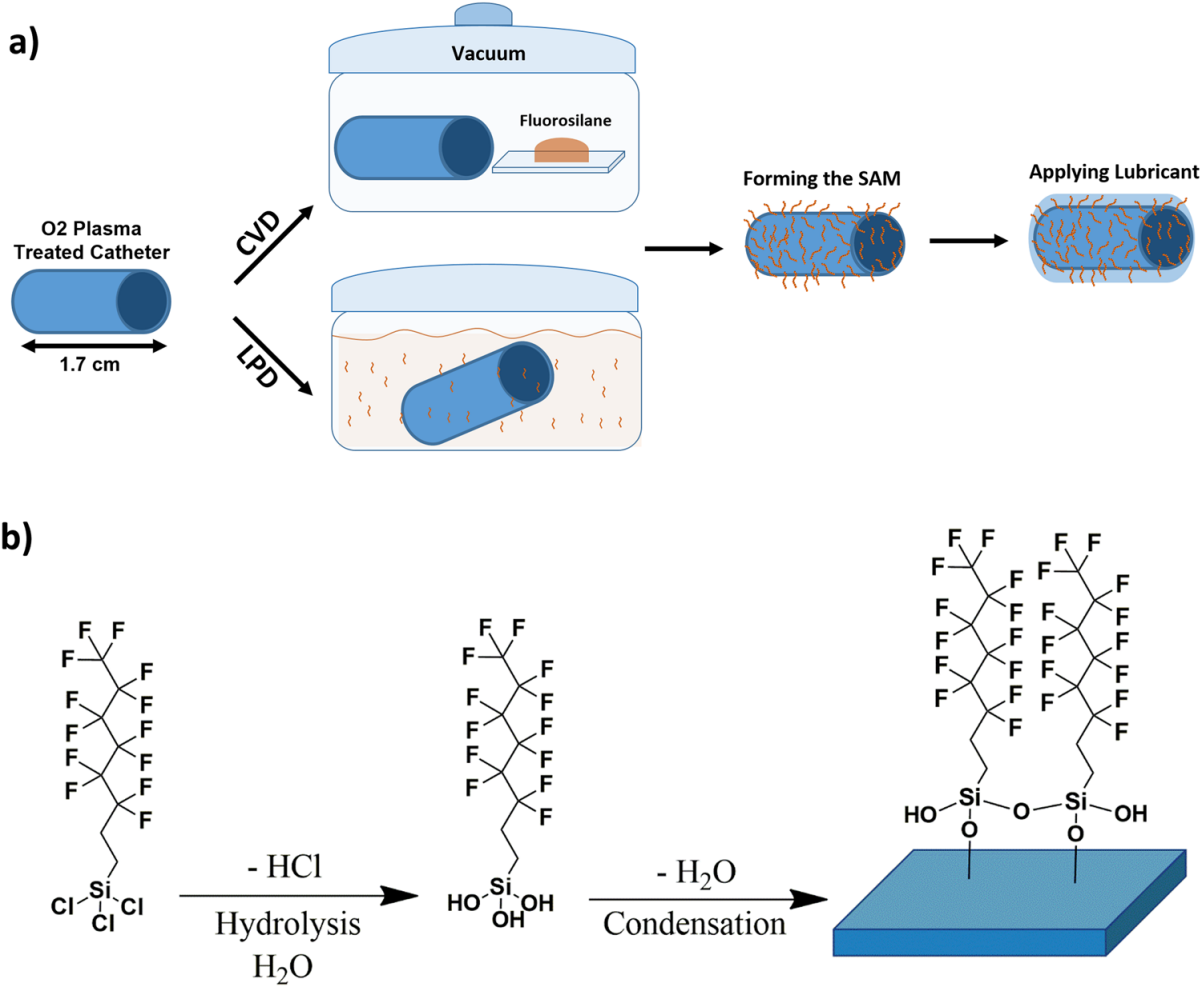
Schematic illustration of the treatment process and the purposed reaction. (**a**) Schematic representation of catheters treated with trichloro (1H, 1H, 2H, 2H-perfluorooctyl) silane (TPFS) through chemical vapor deposition (CVD) and liquid phase deposition (LPD). (**b**) Chemical structure of TPFS and surface functionalization steps of plasma treated catheters with TPFS.

Omniphobic coatings on coronary catheters, composed of a soft polyether amide block on the outer layer were produced using two different chemical modification techniques: 1) The LPD technique, which is the most commonly used method to create omniphobic slippery surfaces^28^, and 2) Our developed CVD method, which is a more efficient, non-invasive and robust process for creating anti-thrombogenic coatings on catheters (Fig. 1a). Catheter segments were oxygen plasma treated and silanized with trichloro (1H, 1H, 2H, 2H-perfluorooctyl) silane (TPFS) using one of the techniques mentioned above (Fig. 1b). In the final step, a biocompatible, FDA approved liquid lubricant such as perfluorodecalin (PFD) or perfluoroperhydrophenanthrene (PFPP) was added to complete the modification process.

### Assessment of surface chemical composition

**Figure 2:**
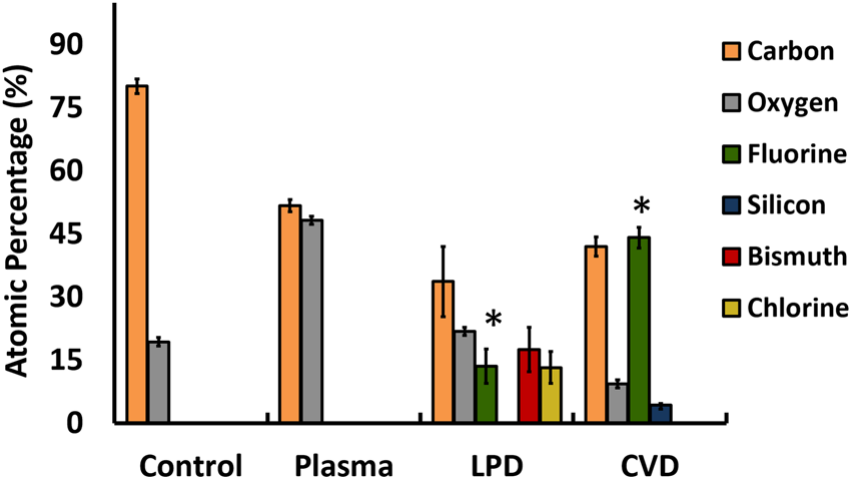
The chemical composition (reported as the percentage atomic concentrations) of the catheter surfaces at different stages of surface modification determined by XPS. Following oxygen plasma, an increase in the oxygen surface concentration was observed and catheters contained up to 50 atom % oxygen. Surfaces treated with LPD had a significantly lower amount of fluorine (about 15 atom %), compared with CVD treated catheters (about 45 atom %). In addition, LPD treated samples showed a large surface concentration of bismuth (>15 atom %) which indicates this treatment method has modified the bulk properties of the catheters. In addition to bismuth, LPD treated catheters had up to 15 atom % of chlorine on their surfaces, an impurity that was not seen on CVD treated catheters. Three samples from each group were analyzed and measurements where performed on four spots on each sample. *Significant difference between the fluorine atom percent when comparing the CVD and LPD treated catheters (*P* < 0.001). The results are presented as means ± S.D.

To examine the changes in the chemical composition of the catheters after oxygen plasma treatment and after CVD or LPD surface modification, X-ray photoelectron spectroscopy (XPS) was performed (Fig. 2). Oxygen plasma treated and silanized catheters showed a significant difference in chemical composition compared with controls. As expected, after oxygen plasma treatment, a high percentage of oxygen (about 50 atom %) was detected on the surface of the catheters, indicating the presence of hydroxyl (OH) groups and consistent with initial activation of the catheter surfaces.

Although fluorine (F) was detected after silanization with both the CVD and LPD method, the fluorine surface concentration was significantly higher with CVD treatment than with LPD treatment (about 45 atom % and 15 atom %, respectively).

Bismuth, the filling used in catheters to render them radiopaque^37^, was detected on the surface of catheters subjected to LPD treatment (>15 atom %). In contrast, bismuth was not detected on the surface of catheters coated using the CVD method. LPD-treated catheters also exhibited chlorine (>10 atom %) on their surface, which was not present on the surface of CVD-treated catheters.

### Contact and sliding angle measurements

**Figure 3:**
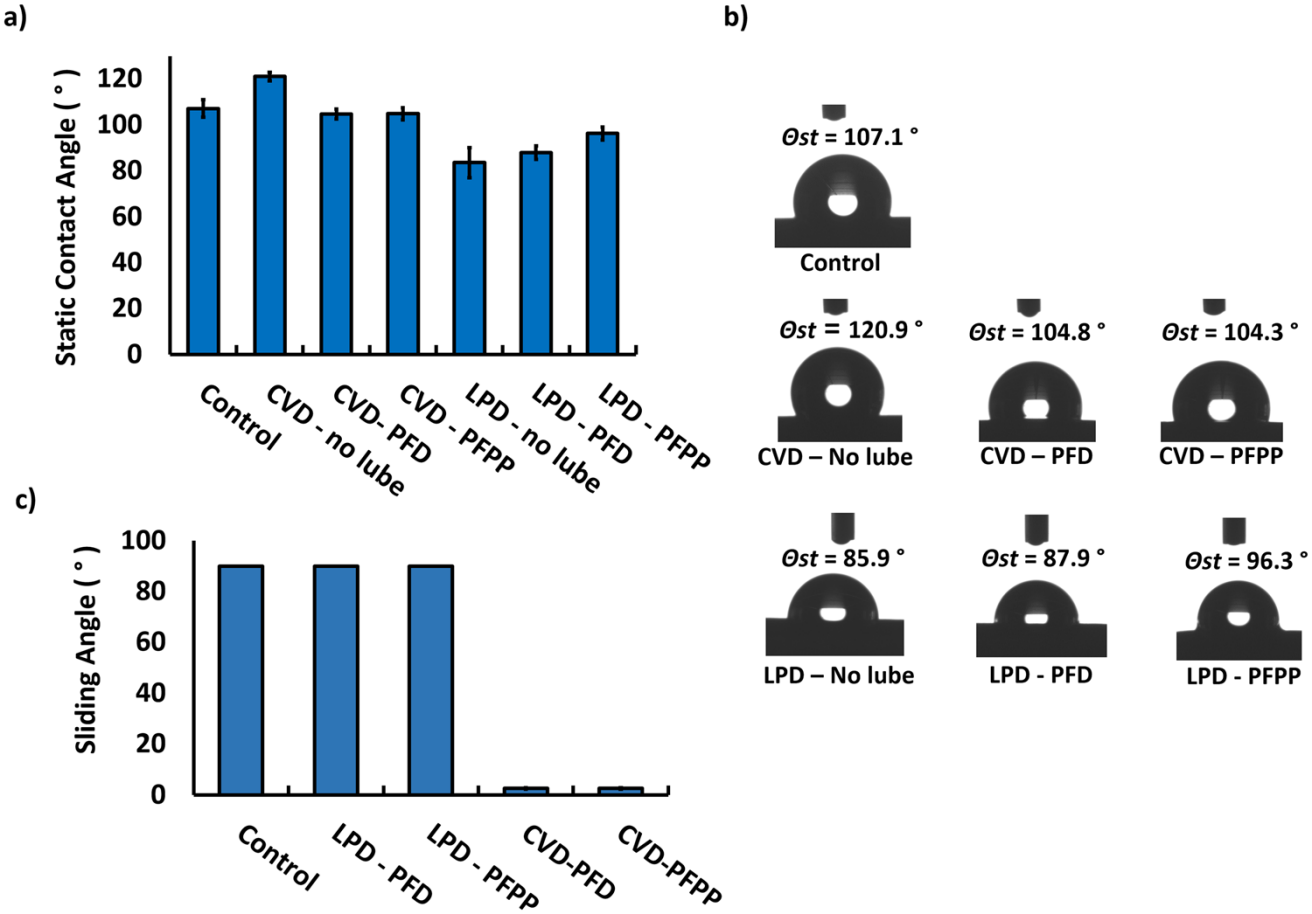
The sliding and contact angle measurements of the control and treated catheters. (**a**) Static contact angle measurement of samples before surface modification (control) and after silanizing the catheters and after adding the lubricant layer. (**b**) Contact angle images of a 5 μL water droplet on the surface of the catheters before and after adding the fluorinated lubricant coating. (**c**) Sliding angle results of the surfaces after adding the lubricant layer. The results are presented as means ± S.D.

To investigate the relative hydrophobicity/hydrophilicity of the control and treated catheters, contact and sliding angle measurements were performed using a 5 µL droplet of deionized water. The sliding angle was defined as the minimum tilting angle required for the droplet to start moving along the catheter surface. A sliding angle of 90 degrees was assigned to droplets that failed to slide at angles of 90 degrees or higher. The static contact angle measurements of the control and treated surfaces before adding the lubricant layer are shown in Fig. 3a and b. Control catheters exhibited a relatively high contact angle (*θ*_*st*_ = 107 ± 4°) indicative of their hydrophobicity. After CVD treatment and before lubricant addition, the water contact angle increased to 121 ± 2°. After the addition of PFD or PFPP lubricant layers to the CVD treated catheters, the water contact angles were lower (104.7 ± 2° and 104.8 ± 2°, respectively).

In contrast to CVD treated surfaces, LPD surfaces had a lower static contact angle (*θ*_*st*_ = 83.6 ± 7°) compared with control surfaces. After adding the lubricants onto these surfaces, the water contact angles remained low. Although the PFPP lubricant increased the contact angle by about 7°, the difference was not significant and the wettability of the surfaces remained high.

Sliding angle measurements of the treated and control surfaces are shown in Fig. 3c. The 5 µL water droplet did not slide on lubricated LPD catheters even with tilting angles higher than 90°, suggesting that these surfaces do not have slippery properties, which is a major characteristic of omniphobic lubricant-infused surfaces. In contrast, with CVD treatment there was a significant increase in liquid repellency compared with the control or LPD catheters as demonstrated by sliding angles as low as 3°. When sliding angle measurements on control and coated catheters were repeated four months later, the results were similar to those obtained on initial measurement (results not shown).

### Sliding angle measurements with whole blood

To investigate catheter-blood interactions and the stability of the coatings, sliding angle measurements were performed with whole blood on catheters that had been treated four months earlier. As seen in Supporting videos 1–3, similar to the results obtained with water, whole blood sliding angles on control and LPD-PFPP treated catheters were greater than 90°. In contrast, CVD-PFPP treated catheters exhibited excellent blood repellency as evidenced by sliding angles less than 3° and immediate sliding of the blood droplet off the catheter surface.

### Effect of catheter modification on plasma clotting times

**Figure 4:**
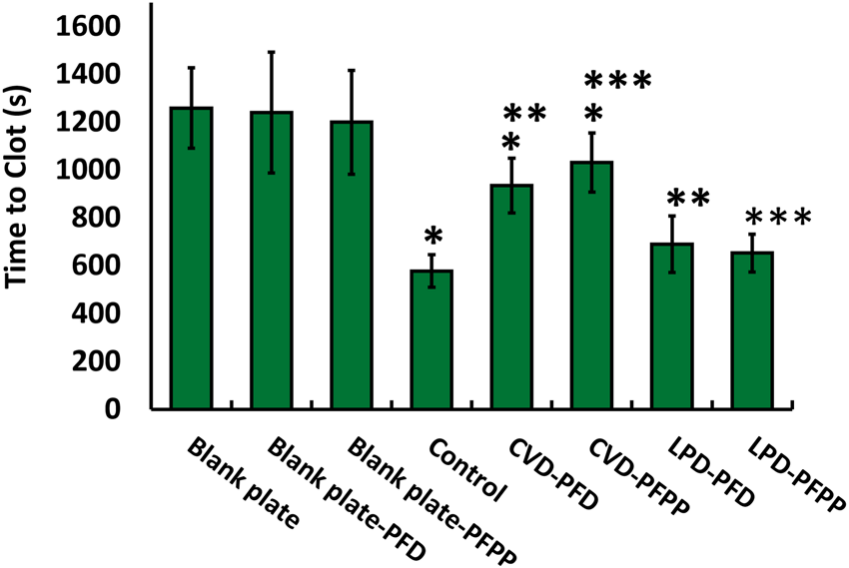
Plasma clotting time when in contact with treated and non-treated catheters and when in blank plates. Catheters were rolled and placed in 96-welplates. After incubating the catheters with the citrated plasma at 37 °C for 5–7 minutes, clotting was initiated by adding 100 µL of CaCl2 solution. Absorbance was calculated over time and clotting time was determined at the time to half-max. The bars represent the means of at least nine repeats from each group. *Significant difference between control catheters *vs*. CVD treated catheters (*P* < 0.05). **, ***Significant difference when comparing the results from the two different treatment types of CVD and LPD (*P* < 0.05). The results are presented as means ± S.D.

Clotting assays were performed to compare the antithrombotic activities of the various coatings. After gently flattening the catheter segments with a plastic roller, they were shaped into rings, placed around the inner walls of the wells of a 96-well plate and saturated with 150 µL of PFPP or PFD lubricant for about 1 min. Empty wells and wells with only lubricant were used as controls. Excess lubricant was removed from the wells and 100 µL aliquots of citrated human plasma were added to wells that did or did not contain catheter segments. The clotting assay was performed as explained in the methods section. As seen in Fig. 4, the average clotting time in wells without a catheter and with no lubricant was 1258 ± 168 s. Empty wells containing PFD or PFPP lubricant had average clotting times of 1239 ± 250 s and 1199 ± 216 s, respectively. Control catheters with no surface modification significantly shortened the clotting time by 2-fold to 577 ± 67 s. Catheters silanized using the CVD method significantly (*P* < 0.001) prolonged the clotting time compared with non-coated catheters to 935 ± 115 s and 1031 ± 123 s, using PFD or PFPP lubricants, respectively. These catheters had the longest clotting times compared with other experimental groups **(**Fig. 4). Both LPD-PFD and LPD-PFPP catheters slightly prolonged the clotting time (689 ± 119 s and 636 ± 87 s, respectively) compared with control catheters, but the differences were not statistically significant. When comparing the results with CVD and LPD catheters, clotting times were significantly (*P < *0.002) longer with the CVD modification method than with the LPD method (Fig. 4).

### Identification of the coagulation pathway activated by modified and unmodified catheters

**Figure 5:**
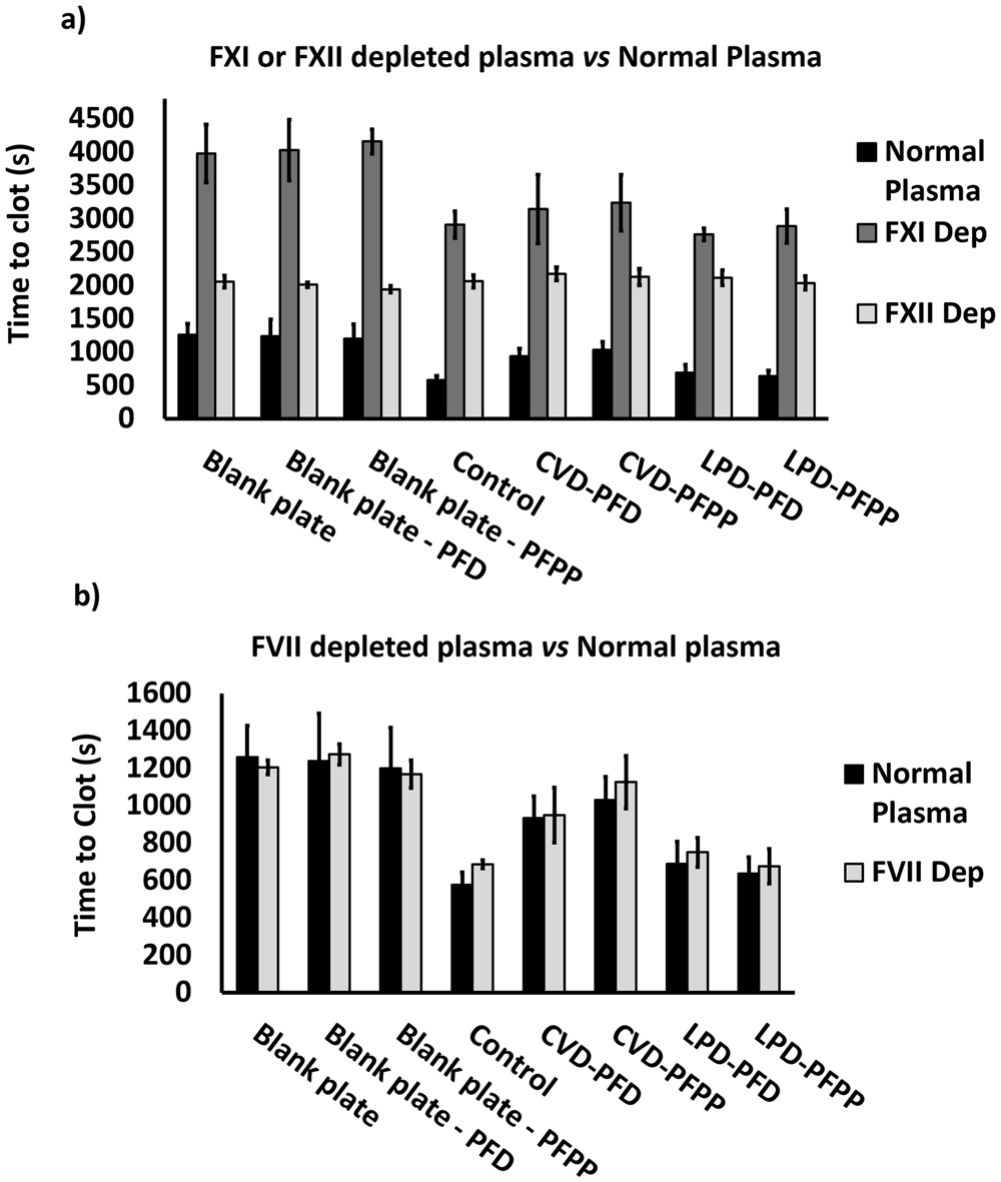
Comparison between the clotting times in normal and FVII, FXII or FXI depleted plasma. Similar to whole plasma clotting assay, catheters were rolled and placed in 96-welplates. After incubating the catheters with depleted plasma at 37 °C for 5–7 minutes, clotting was initiated by adding 100 µL of CaCl_2_ solution. Absorbance was calculated over time and clotting time was determined at the time to half-max. (**a**) Clotting assay in FXI and FXII depleted plasma. Both Control and treated catheters significantly prolong the clotting time in FXI or FXII depleted plasma when comparing the results to normal plasma. (**b**) Clotting assay in FVII depleted plasma. There was no significant difference between the clotting times in FVII depleted plasma when comparing the results to normal plasma. The bars represent the means of at least nine repeats from each group. The results are presented as means ± S.D.

To identify the coagulation pathway involved in catheter-induced clotting, and to determine the effect of the various coatings on such clotting, results from clotting assays performed in control plasma were compared with those in plasma depleted of FXI or FXII, key components of the contact pathway, or FVII, which is the critical component of the extrinsic or tissue factor pathway of the coagulation cascade. Whereas control and modified catheters shortened the clotting time in control or FVII depleted plasma (Fig. 5b), they did not do so in plasma depleted of FXII or FXI (Fig. 5a). This suggests that the procoagulant activity of catheters is dependent on FXII and FXI, but not FVII. Similar to the results in normal plasma, CVD treated catheters shortened the clotting time less than LPD catheters in FVII depleted plasma.

### Protein adhesion and clot formation on the catheter surfaces

**Figure 6:**
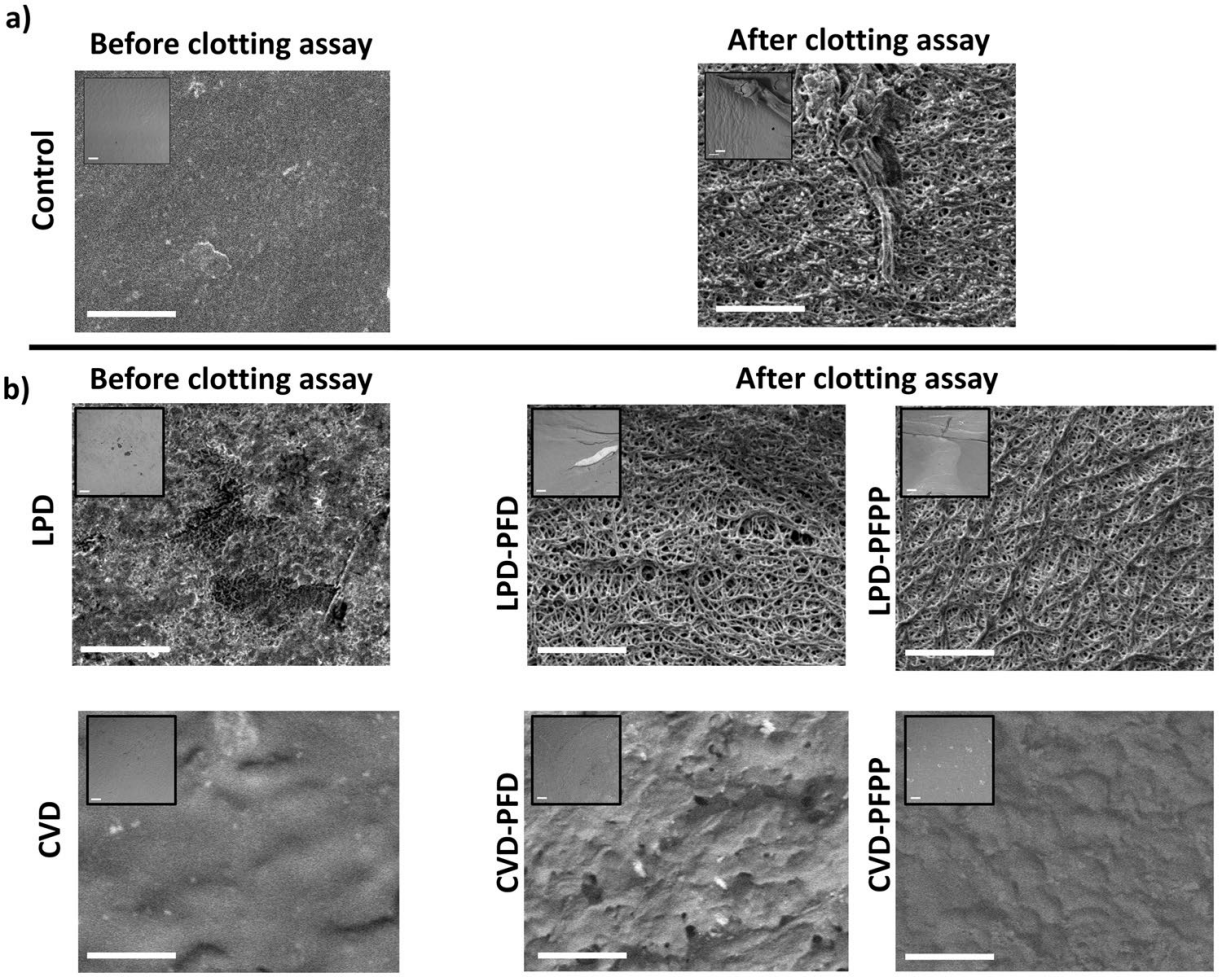
Scanning electron microscopy images of catheters before, after silanization, and after plasma clotting assay. Control (**a**), LPD and CVD treated catheters (**b**) before and after the clotting assay are shown. A uniform smooth silane layer is formed on the catheter surfaces after CVD treating them. In contrast, LPD treated catheters have a rough surface compared with the Controls. Both Control (**a**) and LPD treated catheters (**b**) form a dense protein layer on their surfaces, after the clotting assay, something that is not evident in CVD treated catheters (**b**). The magnification bars are 10 µm on the small images and 1 µm on the larger images.

After coating the catheter surfaces with TPFS using either the CVD or LPD method and after performing the clotting assay in normal plasma, catheter segments were subjected to scanning electron microscopy (SEM) to examine the effect of treatment on the catheter surface topography and to investigate their protein repellency properties. As seen in Fig. 6b, catheters treated with the CVD method had a smooth silane layer on their surface and the surface morphology and roughness were similar to those of control catheters. In contrast, with LPD treatment, there was no evidence of a silane layer and roughness of the surface with etching and exposure of inner layers in some areas was seen under higher magnification.

In addition, as illustrated in Fig. 6a and b, a highly dense protein layer was formed on control and lubricated LPD-treated catheters. In contrast, lubricated CVD-treated catheters exhibited significantly less protein deposition on their surfaces, consistent with the normal clotting assay results.

### Protein deposition and platelet adhesion to catheters in whole blood

**Figure 7:**
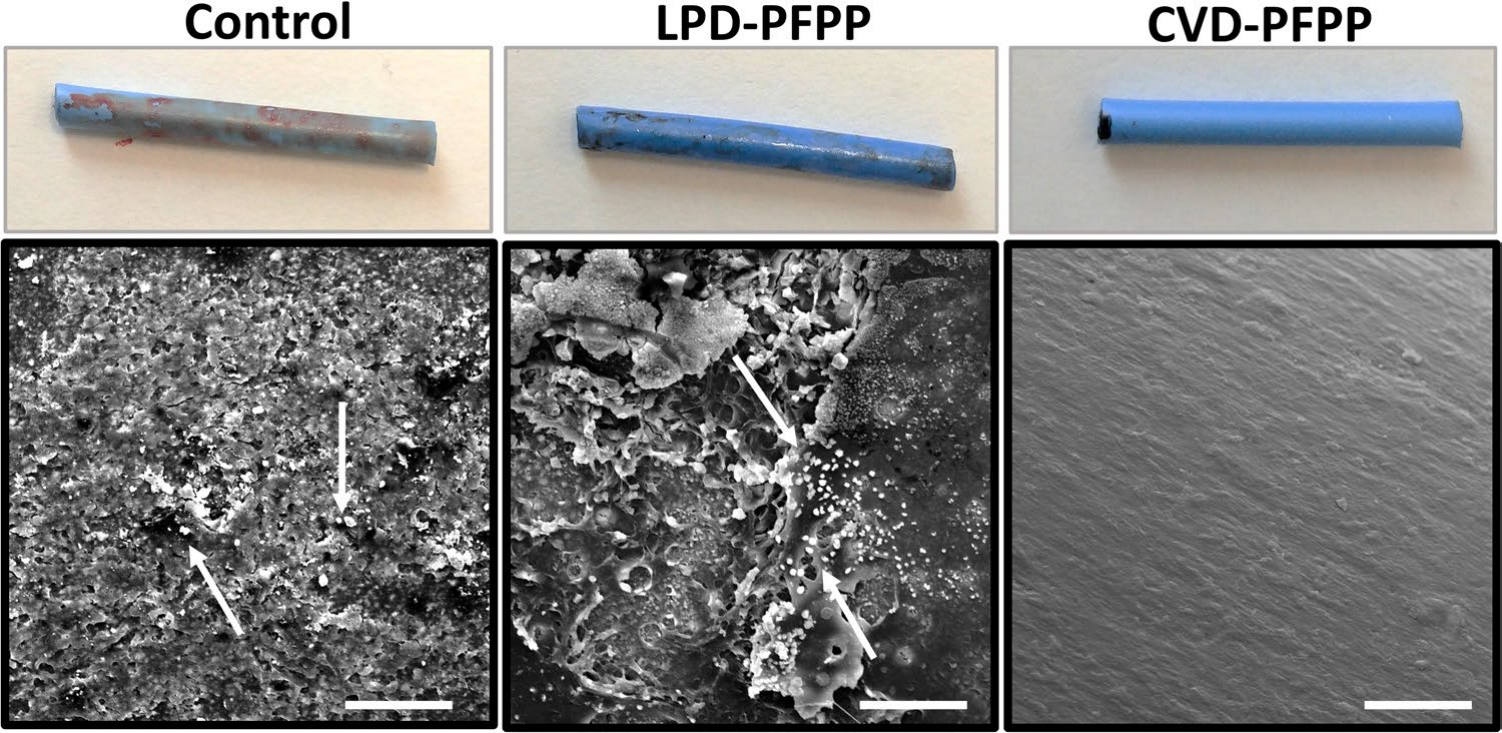
SEM images of catheters incubated with whole blood. Silanized catheters were stored at room temperature and four months after the surface modification procedure, the blood-catheter interaction was investigated. Control, CVD-PFPP and LPD-PFPP catheters were submerged in whole blood for 15 s and images were taken afterwards. Further on, they were washed with PBS, fixed in 4% formaldehyde for 20 mins, and submitted for SEM imaging. Blood clotts were formed on control and LPD-PFPP treated catheters immediatly after being in contact with blood. However, no blood clot formation was seen on CVD-PFPP treated catheters. In addition, platelet adhesion (shown with white arrows) was evident on control and LPD-PFPP treated catheters, while no platelets or protein adhesion was seen on CVD-PFPP treated catheters. The magnification bars are 50 µm.

To assess the stability of the omniphobic slippery coating and the capacity of the coated catheters to resist protein deposition and platelet adhesion, catheter segments that had been treated four months earlier were incubated with whole human blood. Since the PFPP lubricant was superior to PFD lubricant in the clotting assays, CVD and LPD catheters were only lubricated with PFPP in the whole blood experiments. As seen in Fig. 7, after immersing catheter segments in whole blood for 15 s, clot formation was evident on control and LPD-PFPP treated catheters. In contrast, no clot formation was observed on CVD-PFPP treated catheter segments. To further investigate the catheter-blood interaction, blood treated catheter segments were fixed in 4% formaldehyde and submitted for SEM imaging. As seen in the SEM images presented in Fig. 7, a highly dense protein layer was formed on both control and LPD-PFPP catheters, whereas CVD-PFPP catheters showed no protein on their surface. Platelet adhesion was also evident on the control and LPD-PFPP treated catheters, but not on CVD-PFPP catheters.

## Discussion and Conclusions

Thrombosis on blood-contacting medical devices is an ongoing problem. Therefore, there remains a need for surface modification techniques that render such devices more biocompatible^4^. Although LPD is a well described method for producing SAMs of fluorine-based silanes^38^ and is the most widely used technique for producing omniphobic coatings on biomaterials^28,32^, the results of this work show that the CVD method is more efficient and effective than the LPD method for rendering medical grade polymeric catheters less thrombogenic.

A major drawback of the LPD method is the direct exposure of the treated surfaces to the side products produced and released in the liquid solution^38^. Hydrochloric acid, which is the main side product generated during the hydrolysis step of TPFS (Fig. 1b), may damage the polymeric surface of the catheters. Such damage is evident from the XPS and SEM results. With LPD treatment a high atomic concentration of bismuth (>15 atom %) is evident on the surface of the catheters while with CVD treatment no bismuth was detected. Bismuth is introduced to render the catheters radiopaque so that they can be visualized on x-rays during and after insertion^37^. It is likely that hydrochloric acid produced during the liquid treatment process partially degraded the outer polymeric layer of the catheter, thus exposing the bismuth on the surface. This concept is supported by the SEM images, which reveal surface roughness under higher magnification along with etching and exposure of inner layers in some areas in LPD treated but not in CVD treated catheters (Fig. 6b). Although CVD treated catheters were incubated with TPFS for a longer period than LPD catheters (5 h and 1 h, respectively), this did not negatively affect the surface properties of CVD treated catheters. In addition to bismuth, chlorine (>10 atom %) was also present on the surfaces of LPD treated catheters; an impurity not seen on CVD treated surfaces. The presence of chlorine on these catheters could be due to the partial hydrolysis of the Si-Cl bonds and the unsuccessful formation of inner covalent bonds between the silane molecules^39^, suggesting that the LPD method is not as efficient as the CVD method. After treatment with TPFS, the presence of fluorine (F) is expected as a result of formation of the fluorosilane SAM on the catheter surfaces. Although fluorine was detected on both LPD and CVD treated catheters, the fluorine atom concentration on CVD treated samples was significantly higher than that on LPD treated samples (about 45 atom % and 15 atom %, respectively), indicating that the CVD method is a more efficient technique for producing SAM layers of the organosilane. This is further supported by the lower oxygen content on CVD treated samples compared to LPD treated ones, suggesting that with the CVD method, more of the active OH groups were coated with fluorosilane.

Water repellency was greater with the CVD method than with the LPD method as evidenced by lower sliding angles (θ ≤ 5° and θ > 100° respectively). The CVD silanization step transformed the hydrophobic surface of the control catheters to a more hydrophobic surface by increasing the static water contact angle from 107 ± 4° to 121 ± 2°, thereby confirming the presence of the hydrophobic silane coating. In contrast, LPD treated surfaces had a lower contact angle (*θ*_*st*_ = 83.6 ± 7°) compared with the control and CVD treated catheters, confirming the fact that the catheter surfaces were not efficiently coated with a hydrophobic silane layer. In addition, the hydrophobic surface properties were disrupted with the LPD method due to the surface degradation caused by the side products produced during the LPD modification step.

Although the static contact angles decreased in the CVD treated catheters after adding the PFD or PFPP lubricant layer, they were highly water and blood repellant. In contrast, sliding angels were significantly higher with the LPD method (θ > 90°), indicating less omniphobicity and lower water and blood repellency (Fig. 3c, Supporting videos 1–3). This could be due to the etching of the catheter surface and the roughness of the outer layer that occurs with the LPD method. In addition, there is less efficient formation of a SAM layer with the LPD method, which may limit the capacity of the lubricant to completely wet and cover the catheter surface. Therefore, LPD treated catheters showed poorer water and blood repellency compared with CVD treated catheters.

Both lubricants (PFD or PFPP) increased the antithrombotic activity of CVD treated catheters as evidenced by significantly longer clotting times compared with control or LPD treated catheters. The enhanced antithrombotic activity of catheters coated using the CVD method is due to reduced activation of the contact system because this activity is evident in plasma depleted of FVII, which is essential for the extrinsic pathway of coagulation, but not in plasma depleted of FXI or FXII, key components of the contact system. Thus, the findings from these experiments, suggest that modified catheters, similar to unmodified ones, initiate coagulation through the contact pathway and have minimal effect in activating the tissue factor pathway. In both the normal and FVII depleted plasma assays, clotting times were longest with CVD-PFPP catheter segments. PFPP has shown to be more stable than PFD^40^ and has a lower vapor pressure and greater viscosity. Although, immobilized liquid layers modified with PFPP are more durable in open-air environments, this is unlikely to have influenced our results because the lubricated samples were immediately covered with plasma and were maintained in a closed space.

SEM analysis of catheters incubated in plasma or whole blood reveals differences between the CVD and LPD catheters. Due to the omniphobic slippery properties of the CVD treated catheters, no clot formation or platelet adhesion was seen after incubation with whole blood. In contrast, protein deposition and platelet adhesion were observed on the control and LPD treated catheters.

In summary, we reported a simple and biocompatible method for successful production of omniphobic lubricant-infused polymeric medical catheter coatings using CVD of hydrophobic organosilanes. Catheters modified in this manner are less thrombogenic than uncoated catheters and catheters modified using the LPD method.

## Materials and Methods

### Materials

Trichloro (1H, 1H, 2H, 2H-perfluorooctyl) silane (TPFS), perfluoroperhydrophenanthrene (PFPP) and perfluorodecalin (PFD) were purchased from Sigma–Aldrich (Oakville, Canada). Human plasma depleted of FVII, FXI, or FXII was purchased from Affinity Biologicals (Ancaster, Canada). Coronary catheters (Medtronic, Minneapolis, USA) composed of a soft hydrophobic polyether amide block on the outer layer and a thin walled polytetrafluoroethylene (PTFE) tube on the luminal side^41^ were generously provided by S. Gracie. Whole blood and pooled citrated plasma was generated from blood samples collected from healthy donors as previously described^42^. All donors provided signed written consent. All procedures were approved by the McMaster University Research Ethics Board.

### Oxygen plasma treatment of catheter segments

Prior to silanizing the catheters, they were cut into 1.7 cm segments, a length chosen to enable placement in the wells of 96-well polystyrene plates (Evergreen Scientific). Segments were then vertically fixed on plastic petri dishes, placed in an oxygen plasma cleaner (Harrick Plasma Cleaner, PDC-002, 230 V) and exposed to high-pressure oxygen plasma for 2 minutes to functionalize their surfaces and to enable reaction with TPFS.

### Preparation of silanized catheters using CVD

After removing the oxygen plasma-treated catheters from the plasma cleaner, they were immediately placed in a desiccator connected to a vacuum pump and two droplets (200 µL) of TPFS were added in a separate petri dish on the side of the catheters. The vacuum pump was turned on and once a pressure of −0.08 MPa was achieved, the exit valve was closed and CVD of the silane onto the catheters was initiated. The silanization reaction was carried out for 5 hours at room temperature. After the CVD step, catheters were removed from the desiccator and placed in an oven at 60 °C for a minimum of 12 h in order to complete the reaction. After removing the catheters from the oven, CVD-modified catheters were placed under vacuum for 30 mins with an open exit valve to ensure removal of non-bonded silanes from the surface.

### Preparation of silanized catheters using LPD

Catheters were oxygen plasma treated as described above and then immediately incubated in a 20 mL glass vial containing TPFS in anhydrous ethanol solution (5% (v/v)). The solution was stirred with a small magnetic stir bar for 1 h at room temperature. LPD-treated catheters were removed from the solution and then washed with 100% anhydrous ethanol followed by deionized water and ultimately with 70% ethanol. Washed catheters were left to dry at room temperature and then placed in the oven at 60 °C overnight. Similar to CVD treated catheters, after removing the LPD treated catheters from the oven, they were placed under vacuum for 30 mins with an open exit valve to ensure removal of non-bonded silanes from the surface.

### Applying fluorinated lubricants on silanized catheters

As a final step, and before preforming different measurements, CVD and LPD treated catheters were submerged into fluorinated lubricants in order to complete the surface modification. Two types of fluorinated lubricants were used: perfluoroperhydrophenanthrene (PFPP) and perfluorodecalin (PFD).

### X-ray photoelectron spectroscopy (XPS)

XPS was used to assess the surface chemical composition of the catheters before and after each treatment step. For each condition, three catheter segments were subjected to XPS analysis, measurements were taken from four distinct sites on each segment, and means were determined. XPS spectra were recorded using a Physical Electronics (PHI) Quantera II spectrometer equipped with an Al anode source for X-ray generation and a quartz crystal monochromator was used to focus the generated X-rays (BioInterface Institute, McMaster University). The monochromatic Al K^−α^ X-ray (1486.7 eV) source was operated at 50 W 15 kV with a system base pressure no higher than 1.0 × 10^−9^ Torr and an operating pressure that did not exceed 2.0 × 10^−8^ Torr. A pass energy of 280 eV was used to obtain survey spectra and spectra were obtained at 45° take off angles using a dual beam charge compensation system for neutralization. The raw data were analyzed using the instrument software and the atom percentages of carbon, oxygen, fluorine, bismuth, silicon and chlorine were calculated.

### Contact and sliding angle measurements

Contact and sliding angles of the treated and non-treated catheters were measured using a droplet of deionized water (5 µL). Water sessile drop contact angle measurements were performed at room temperature before and after each modification step using a Future Digital Scientific OCA20 goniometer (Garden City, NY), which was calibrated prior to each measurement. Sliding angles were measured using a custom-made goniometer. Immediately prior to testing, silanized samples coated with PFPP or PFD were placed on the calibrated goniometer. A droplet of deionized water (5 µL) was placed on the catheter surface and the sample was gently tilted until the droplet started to move. The sliding angle was defined as the minimum tilting angle required for droplet movement. A sliding angle of 90 degrees was assigned to droplets that failed to slide at angles of 90 degrees or higher. Measurements were made in triplicate on three different catheter segments and means were determined.

### Antithrombotic activity of modified catheters

Clotting assays were performed to compare the antithrombotic activities of the various coatings. After flattening the catheter segments with a plastic roller, they were shaped into rings, placed around the inner walls of the wells of a 96-well plate and saturated with 150 µL of PFPP or PFD lubricant for about 1 min. Excess lubricant was removed and 100 µL aliquots of citrated human plasma were added to wells that did or did not contain catheter segments. After incubating the plate for 5–7 minutes at 37 °C, clotting was initiated by adding HEPES (100 µL of 20 mM, pH 7.4) containing CaCl_2_ (1 M) to each well, yielding a final CaCl_2_ concentration of 25 mM^7,43^. Clot formation was assessed by monitoring absorbance at 405 nm at 10-sec intervals for 60 min in kinetic mode using a SPECTRAmax plate reader (Molecular Devices). Clotting times were defined as the time to reach half-maximal absorbance as calculated by the instrument software from plots of absorbance versus time. The same procedure was repeated in FVII, FXI, or FXII depleted plasma, except that absorbance was monitored over 3 hours to account for the longer clotting times.

### Whole human blood experiments

Treated catheters were stored at room temperature and four months later, the stability of their coating was investigated by performing sliding angle measurements and catheter-blood interaction experiments using whole human blood. Sliding angle measurements with blood were performed according to the procedure described above (contact and sliding measurements).

To investigate catheter-blood interactions, control and treated catheters were submerged in whole human blood for 15 s. Catheters were then washed with PBS, fixed in 4% formaldehyde for 20 min and stored at room temperature in PBS until SEM analysis. Using SEM, the extent of clot formation and platelet adhesion on the catheter surfaces was evaluated.

### Scanning Electron Microscopy (SEM)

Catheter segments were washed three times, fixed in 4% formaldehyde in PBS for 2 hours, washed with PBS (0.1 M) and sputter-coated with a 4 nm thick platinum coating. SEM imaging (JSM- 7000 F) was performed in secondary electron image (SEI) mode with voltages of 1.0 kV at 10,000x magnification or 2.0 kV at 1000x magnification.

### Statistical Analysis

Data are presented as means ± S.D. In the control and depleted plasma clotting assays, each experimental condition was repeated at least nine times. For all other studies, experiments were repeated at least three times. One-way analysis of variance (ANOVA) followed by post hoc analysis using Tukey’s test was performed to assess statistical significance. For all comparisons, *P* values less than 0.05 were considered statistically significant.

### Data availability statement

The datasets generated during and/or analysed during the current study are available from the corresponding author on reasonable request.

## Electronic supplementary material

Control Catheter

LPD Catheter

CVD Catheter
